# The Accuracy of Three-Dimensional Soft Tissue Simulation in Orthognathic Surgery—A Systematic Review

**DOI:** 10.3390/jimaging10050119

**Published:** 2024-05-14

**Authors:** Anna Olejnik, Laurence Verstraete, Tomas-Marijn Croonenborghs, Constantinus Politis, Gwen R. J. Swennen

**Affiliations:** 1 Division of Maxillofacial Surgery, Department of Surgery, AZ Sint-Jan, Ruddershove 10, 8000 Bruges, Belgium; 2 Maxillofacial Surgery Unit, Department of Head and Neck Surgery, Craniomaxillofacial Center for Children and Young Adults, Regional Specialized Children’s Hospital, ul. Zolnierska 18A, 10-561 Olsztyn, Poland; 3 Department of Oral and Maxillofacial Surgery, University Hospitals Leuven, 3000 Leuven, Belgium

**Keywords:** orthognathic surgical procedures, orthognathic surgery, three-dimensional image, patient simulation, data accuracy

## Abstract

Three-dimensional soft tissue simulation has become a popular tool in the process of virtual orthognathic surgery planning and patient–surgeon communication. To apply 3D soft tissue simulation software in routine clinical practice, both qualitative and quantitative validation of its accuracy are required. The objective of this study was to systematically review the literature on the accuracy of 3D soft tissue simulation in orthognathic surgery. The Web of Science, PubMed, Cochrane, and Embase databases were consulted for the literature search. The systematic review (SR) was conducted according to the PRISMA statement, and 40 articles fulfilled the inclusion and exclusion criteria. The Quadas-2 tool was used for the risk of bias assessment for selected studies. A mean error varying from 0.27 mm to 2.9 mm for 3D soft tissue simulations for the whole face was reported. In the studies evaluating 3D soft tissue simulation accuracy after a Le Fort I osteotomy only, the upper lip and paranasal regions were reported to have the largest error, while after an isolated bilateral sagittal split osteotomy, the largest error was reported for the lower lip and chin regions. In the studies evaluating simulation after bimaxillary osteotomy with or without genioplasty, the highest inaccuracy was reported at the level of the lips, predominantly the lower lip, chin, and, sometimes, the paranasal regions. Due to the variability in the study designs and analysis methods, a direct comparison was not possible. Therefore, based on the results of this SR, guidelines to systematize the workflow for evaluating the accuracy of 3D soft tissue simulations in orthognathic surgery in future studies are proposed.

## 1. Introduction

In orthognathic surgery, two-dimensional (2D) planning programs based on lateral cephalograms and clinical profile photographs have been used for decades in clinical practice. However, the use of 2D lateral cephalograms is prone to analysis bias due to the superimposition of three-dimensional (3D) anatomical structures [1]. The main limitations of 2D planning programs are the simplifications of the algorithms in the simulation of soft tissue changes, because they use fixed hard-to-soft-tissue ratios for the prediction of soft tissue results, and they are unable to predict changes in the transverse plane [2].

The introduction of 3D planning software based on cone beam computed tomography (CBCT) and patients’ high aesthetic demands have led to a paradigm shift in the philosophy of 3D surgical planning [3], where “the bite indicates a problem—the face indicates how to treat the ‘bite’” [4]. During the surgical procedure, surgeons do not directly operate on facial soft tissues but rely on their passive change after the repositioning of the bony segments [5]. While virtual treatment planning (VTP) of bony movements is predictable [6,7], currently, a reliable algorithm for predicting the postoperative facial soft tissue appearance does not exist [8,9].

The application of 3D soft tissue simulation extends beyond mere visualization, offering valuable insights into the aesthetic implications of orthognathic surgery and facilitating effective communication between the surgeon, the orthodontist, and the patient [10]. This collaborative approach fosters informed decision making and clearer understanding of the proposed treatment plan and realistic expectations for the surgical outcome.

Before applying 3D simulation software in routine clinical practice, both qualitative and quantitative validation are required [11] to evaluate whether the simulations are accurate representations of the expected soft tissue changes [10]. Hence, a series of studies have been published evaluating the accuracy of 3D soft tissue simulation by comparing it to the actual postsurgical soft tissue outcome. However, whereas the superimposition and measurement techniques of planned and postoperative images in the 2D environment were well established many years ago, the 3D environment is much more complex, with significant inconsistency, and there is no consensus regarding the ideal assessment method [7].

The objective of this study was to systematically review the literature on the accuracy of 3D soft tissue simulation in orthognathic surgery. Based on the insights gained from this review, we propose standardizing the methodology for evaluating the accuracy of 3D soft tissue simulation in orthognathic surgery. This standardized approach aims to minimize the risk of errors and analysis bias in future studies.

## 2. Materials and Methods

This study was planned based on the Population Intervention Comparison Outcome Study design (PICOS) format, as presented in Table 1.

On 13 January 2023, the Web of Science, PubMed, Cochrane, and Embase databases were used for the literature search. Specific search strategies using the search terms “soft tissue” and “orthognathic surgery” were performed in each database in collaboration with a professional librarian. The full search string for each database is included in Supplementary Materials Table S1. There were no restrictions in the search strategy regarding the year of publication. No additional search of the gray literature was performed. On 20 April 2024, before finishing the manuscript, the search was repeated to detect any new studies that could also be included. The inclusion and exclusion criteria are listed in Table 2.

**Figure 1:**
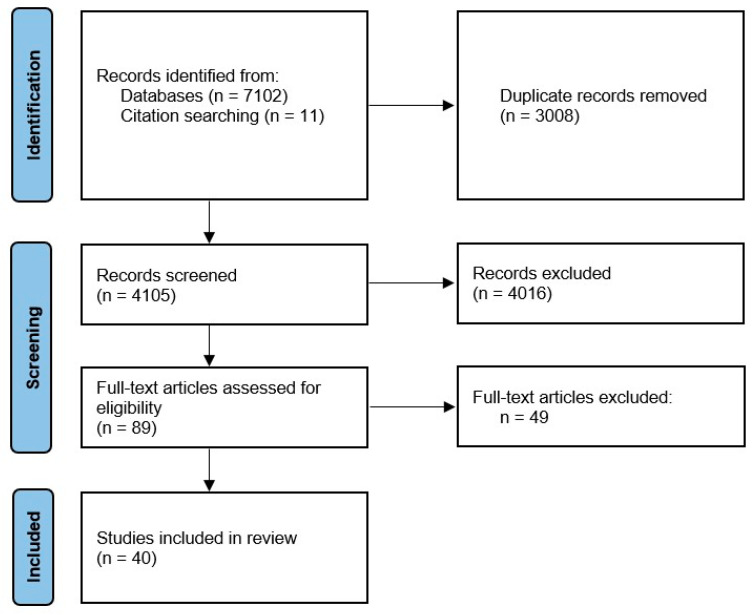
PRISMA 2020 flow diagram.

This review was registered in the International Prospective Register of Systematic Reviews (PROSPERO: CRD42020130214). The Preferred Reporting Items for Systematic Reviews and Meta-Analysis (PRISMA) statement was used for selecting studies (http://www.prisma-statement.org/ accessed on 20 April 2024) [12]. The PRISMA flow diagram can be found in Figure 1.

The Covidence (Veritas Health Innovation, Melbourne, Australia) tool was used for the screening phase and full text review. The eligibility of the studies was checked independently by two junior authors. In case of disagreement, the study was discussed with the senior author. Studies that did not meet the eligibility criteria were excluded from further analysis.

Qualitative and quantitative data were independently extracted from the studies using a standardized form. The following data were registered: year of publication, first author, study design, sample size, mean age (years), gender, type of facial deformity, type of surgery, medical imaging technique (CBCT/MSCT, 3D photographs), image acquisition protocol, software package and/or algorithm, type of rigid registration method for soft tissue evaluation, method of analysis, fixed point of accuracy, and results.

### Study Quality and Risk of Bias Assessment

The revised Quadas2 tool for assessing risk of bias and applicability in systematic reviews for diagnostic accuracy-related studies was used in this study. This tool comprises five domains: patient selection, index test, reference standard, flow, and timing. It allows for a transparent rating of the bias and applicability of included studies. Each domain is assessed in terms of risk of bias, and the first three domains are also assessed for applicability concerns [13]. The assessment was carried out independently by two junior authors. In case of disagreement, the issue was discussed with the senior author. 

## 3. Results

The initial search yielded 7113 articles, which were processed through abstract screening, from which 89 articles were selected for full text reading. Finally, 40 articles fulfilled the inclusion and exclusion criteria after the full text review. 

The studies included in this review assessed the accuracy of 3D soft tissue simulation by comparing the actual postoperative soft tissue outcome to the 3D soft tissue simulation based on the VTP. For the VTP and 3D soft tissue simulation, various commercially available programs were used, as well as advanced software platforms that are limited in use to engineers only (Table 3).

Descriptive data on the included studies are presented in Table 4. While this paper presents an abridged table, an equivalent but complete table is included in Supplementary Materials Table S2. The papers included in this SR were assessed in terms of risk of bias, as described above, and these assessments are presented in Supplementary Materials Table S3.

In the 40 included studies, the sample size varied from 3 to 100 patients. A total of 1021 simulations were evaluated. Among the studies, there was variability in the types of facial deformities that were included in the study sample: 10 studies [10,15,16,18,19,29,30,31,36,37] included patients with skeletal class III malocclusion, 3 studies [22,32,33] included patients with skeletal class II malocclusion, 12 studies [3,9,17,24,25,26,34,35,38,39,40,41] included heterogenous groups, and 1 study [20] only included facial asymmetry. In 14 studies [8,11,14,21,23,27,28,42,43,44,45,46,47,48], information about the type of deformity was missing or unclear.

Three-dimensional soft tissue simulations of different orthognathic surgical procedures were described. Six studies [15,16,19,30,37,45] evaluated the simulation of Le Fort I osteotomy, two studies [22,33] evaluated that of mandibular osteotomy, fifteen studies [9,10,14,17,21,23,27,28,29,31,32,34,36,47,48] evaluated that of bimaxillary osteotomy (with or without genioplasty), and in thirteen studies [3,11,18,20,24,25,26,38,39,40,41,44,46], different types of procedures were considered. In four studies [8,35,42,43], information about the surgical procedure was not reported or unclear.

Multi-slice computed tomography (MSCT) records were used in 12 studies [3,8,9,27,29,32,35,41,42,43,46,48], whereas in 23 studies [10,11,14,15,16,17,19,20,21,22,23,24,25,28,30,31,33,34,37,38,39,40,45,47] CBCT records were utilized. Three other studies [18,26,44] used both MSCT and CBCT records. One study combined CBCT and MRI records [47]. In one study [36], 3D photographs and 2D lateral cephalograms were combined, and in thirteen studies [3,9,10,11,15,31,33,34,38,39,40,41,44], both 3D photographs and MSCT/CBCT records were taken.

The time interval for postoperative image acquisition varied from 3 to 6 months in seven studies [3,11,24,25,26,34,46] to at least 4 months in one study [18], exactly 6 months in seven studies [27,28,29,35,38,44,47], at least 6 months in ten studies [9,10,15,21,22,23,31,32,41,45], 6–12 months in five studies [14,17,19,20,37], 12 months in two studies [16,33], and 11–15 months in one study [30]. In seven studies [8,36,39,40,42,43,48], information about the postoperative image acquisition time interval was not reported. 

**Table 4:** Descriptive data on the studies included in the review.

Year, First Author	Sample Size	CBCT/MSCT ^a^	Three-Dimensional Photo ^a^	Software Package and/or Algorithm	Type of Surgery	Results ^b^
2004, Chabanas [42]	3	MSCT **	No	FEM	NR	ME range: 1–1.5 mm, MaxE: 3–6 mm
2007, Mollemans [3]	10	MSCT **	Yes	(1) Linear FEM; (2) non-linear FEM; (3) MSM; (4) MTM	TRIMAX, BIMAX, BSSO, BSSO + Ch, LFI + Ch	Average median distance for MTM: 0.60 mm, FEM: 0.60 mm, MSM: 0.64 mm, NFEM: 0.63 mm; average 90th percentile distance for MTM: 1.48 mm, FEM: 1.51 mm, MSM: 1.67 mm, NFEM: 1.71 mm; highest accuracy: FEM and MTM
2007, Marchetti [46]	25	MSCT	No	VISU system	LFI, BSSO, LFI + Ch, BSSO + Ch, BIMAX, TRIMAX	Error < 2 mm in 80% (20 of 25) of the patients
2010, Bianchi [28]	10	CBCT	No	SurgiCase CMF Pro v.1.2	BIMAX/TRIMAX	ME: 0.94 mm; error < 2 mm in 86.8% of the simulations; 90th percentile: 2.24 mm; 95th percentile: 2.81 mm
2010, Ulusoy [43]	6	MSCT **	No	Dynamic volume spline	BIMAX *	ME: 1.8 mm
2011, Centenero [26]	16	MSCT/CBCT	No	SimPlant ProOMS v.10.1	BIMAX, TRIMAX, BSSO + Ch	5 of 8 ST measurements: high degree of correlation; 3 measurements: medium degree of correlation
2011, Marchetti [29]	10	MSCT	No	SurgiCase CMF Pro v.1.2	BIMAX, TRIMAX	ME: 0.75 +/− 0.78 mm; error < 2 mm in 91% of the simulations; 90th percentile: 1.94 mm; 95th percentile: 2.47 mm
2013, Schendel [38]	23	CBCT	Yes ^c^	3dMDVultus—MSM	LFI, BSSO, Ch	Entire face ME: 0.27 mm, ComR: 1.10 mm, ComL: 0.99 mm, Pog: 0.79 mm
2013, Shafi [19]	13	CBCT	No	Maxilim v.2.2.0—MTM	LFI	ME: 0.97 mm; all anatomical regions with error significantly < 3.0 mm, exception UL error: 2.73 +/− 1.72; overprediction of UL
2013, Nadjmi [11]	13	CBCT ** lat ceph	Yes **	(1) 2D Dolphin v.10—fixed hard-tissue-to-soft-tissue ratios; (2) Maxilim—MTM	LFI, LFI+Ch, BIMAX, TRIMAX	Dolphin range of error in horizontal position: −1.41 to 1.20 mm, in vertical position: −1.85 to 1.55 mm; Maxilim range of error in horizontal position: −1.60 to 1.50 mm, in vertical position: −4.25 to 2.42 mm. No statistical differences between software, exception SA in Maxilim
2014, Terzic [44]	13	MSCT/CBCT	Yes ^c^	3dMDvultus v.2.2.0.8—MSM	BSSO, BSSO+Ch, BIMAX, TRIMAX	ME for the upper part: +0.27 mm, the lower part: –0.64 mm; in the lower part, error < +/− 1 mm 26.9%, >+/− 1 mm 73.1%, >+/− 2 mm 49.5%, and >+/− 3 mm 29.8%
2014, Nadjmi [24]	20	CBCT	No	Maxilim—MTM	BSSO, BIMAX, TRIMAX	ME: 1.18 mm; 84% of errors between −2 mm and +2 mm
2015, Ullah [37]	13	CBCT	No	3dMDVultus v.2.2.0—MSM	LFI	ME: 0.92 mm (0.3–2.4 mm); 90th percentile from 0.65 mm (chin) to 1.17 mm (UL); ME significantly < 3 mm; the 95% CI in all regions < 2 mm
2015, Khambay [45]	10	CBCT **	No	3dMDvultus v.2.2.0—MSM	LFI	ME for 95th percentile: 0.98–0.56 mm, for 90th percentile: 0.91–0.50 mm; error < 2 mm: 94.4%—85.2% points; RMS error: 2.49–0.94 mm; RMS difference for all measurements: 1.3 mm
2015, Nam [27]	29	MSCT	No	Simplant Pro	BIMAX, TRIMAX	ME in all landmarks: 2.03 mm; error < 2 mm: 52.8%; absolute error values in the *x*-axis: 0.73 mm, *y*-axis: 1.39 mm, *z*-axis 0.85 mm; error significantly > 2 mm: ChR, ChL, LL, Pog; MaxE: 2.38 mm in ChL, MinE: 0.84 mm in pronasale
2015a, Liebregts [23]	60	CBCT	No	Maxilim—MTM	BIMAX	Landmarks: MaxE at LI: 3.1 +/− 1.4 mm, MinE at SN: 1.5 +/− 0.6 mm; surfaces: entire face ME: 0.81 +/− 0.22 mm, for UL: 1.2 +/− 0.6 mm, LL: 1.4 +/− 0.5 mm, chin: 1.1 +/− 0.6 mm; error equal to or < 1 mm: 83.3%, < 2 mm: 100%; ME among patients who had a V-Y closure was significantly smaller than those without a V-Y closure
2015b, Liebregts [22]	100	CBCT	No	Maxilim v.2.2.2.1—MTM	BSSO	Landmarks: ME at SN: 1.1 +/− 0.5 mm, at LS: 1.5 +/− 0.7 mm, at LI: 2.0 +/− 1.0 mm, at sublabial: 1.7 +/− 1.1 mm, at Pog: 1.5 +/− 0.9 mm; surface: entire face ME: 0.9 +/− 0.3 mm; error equal to or <2 mm: 100%, <1 mm: 78%; ME for UL: 0.9+/− 0.5, LL: 1.2+/− 0.5, and chin: 0.8 +/− 0.5 mm; average absolute error less or equal to 2 mm for UL: 98%, for LL: 94%, and for the chin: 97%
2015, Van Hemelen [25]	31	CBCT	No	Maxilim—MTM	BSSO, BSSO + Ch, LFI, LFI + Ch, BIMAX, TRIMAX	ME in the horizontal direction: 1.48 mm, in the vertical direction: 1.46 mm
2016, Liebregts [21]	60	CBCT	No	Maxilim—MTM	BIMAX	ME: 1.0 +/− 0.9 mm in alar width
2016, Resnick [15]	7	CBCT	Yes ^c^	Dolphin 3D v.11.8—sparse landmark-based algorithm	LFI	ME: 2.91 +/− 2.16 mm, for midline points: 1.66 +/− 1.82 mm, for lateral points: 3.84 +/− 1.92 mm; 2 (33%) midline points with error > 2 mm (SN, SA), 6 (75%) lateral points > 2 mm; ME at NLA: 8.1 +/− 5.6 degrees
2017, Kim [9]	40	MSCT **	Yes ^c^	FEM with the mucosa sliding effect	BIMAX, TRIMAX	Quantitative: entire face ME: 1.1 +/− 0.3 mm, UL: 1.2 +/− 0.7 mm, LL: 1.5 +/− 0.7 mm, chin: 1.3 +/− 0.7; qualitative: 80% (32/40) clinically acceptable
2021, Kim [41]	35	MSCT **	Yes ^c^	FEM with the sliding effect of the lip and the mucosa	BSSO, BIMAX, TRIMAX	Quantitative: entire face ME: 1.03 +/− 0.30 mm, UL: 0.86 +/− 0.36 mm, LL: 1.10 +/− 0.41 mm, chin: 1.08 +/− 0.51 mm; qualitative: improvement in lips compared with previous FEM methods
2017, Mundluru [20]	13	CBCT	No	Maxilim—MTM	BIMAX, BSSO, BSSO+Ch	Underprediction of ST changes; signed ME from −0.55 to 0.43 mm; absolute ME from 0.6 to 1.3 mm
2018, Holzinger [35]	16	MSCT	No	SOTIRIOS	NR—surgery first	ME: 1.46 +/− 1.53 mm; 50% < 1.03 mm, 80% < 2.20 mm, and 95% up to 4.34 mm
2019, Knoops [16]	7	CBCT	No	(1) Dolphin 3D v.11.95—sparse landmark-based algorithm; (2) ProPlan CMF v.3.0.1—FDM; (3) PFEM	LFI	RMSDolphin = 1.8 +/− 0.8 mm, RMSPro-Plan = 1.2+/− 0.4 mm, and RMSPFEM = 1.3+/− 0.4 mm; average percentage of points < 2 mm: PDolphin = 83+/− 12%, PProPlan = 91+/− 9%, and PPFEM = 88+/− 10%; better results for ProPlan and PFEM compared to Dolphin
2019, Elshebiny [14]	20	CBCT	No	Dolphin 3D v.11.9—sparse landmark-based algorithm	BIMAX/TRIMAX	Statistically significant differences in 2 angular measurements (FNA and NLA) and in 3 linear measurements (SA, UL length, and subalar width)
2021, Cunha [32]	16	MSCT **	No	OrtogOnBlender-OOB—MSM	BIMAX/TRIMAX	ME for all landmarks < 2 mm, entire face ME: 1.07 mm; MaxE: ChR, ChL, and SB
2021, Willinger [18]	19	MSCT/CBCT **	No	(1) IPS Case Designer—MTM; (2) Dolphin 3D v.11.95—sparse landmark-based algorithm	Modified IQLFIIO +/− BSSO	IR level: Dolphin ME: 2.90 +/− 2.1 mm, IPS ME: 1.70 +/− 1.3 mm; SF level: Dolphin ME: 3.57 +/− 2.0 mm, IPS ME: 1.34 +/− 0.9 mm; Li level: Dolphin ME: 2.48 +/− 1.9 mm, IPS ME: 2.25 +/− 1.6 mm; MaxE for Dolphin at SF level
2021, Tanikawa [36]	72	No—lat ceph	Yes	Geometric morphometric methods (GMMs), DL	BIMAX	System error: 0.89 ± 0.30 mm; MaxE of 0.8–1.2 mm in the nasal ala, chin, corner of the mouth; total success rate at <1 mm: 54%, and at <2 mm: 100%
2021, ter Horst [33]	14	CBCT	Yes ^c^	DL; IPS CaseDesigner—MTM	BSSO	DL-based: lower face ME: 1.0 +/− 0.6 mm, simulations with MaxE of 1 mm: 64.3% and of 2 mm: 92.9%; RMS: 1.2 +/− 0.6 mm; ME: for LL 1.1 +/− 0.9 mm; for the chin: 1.4 +/− 0.9 mm. MTM-based: lower face ME: 1.5 +/− 0.5 mm, simulations with MaxE of 1 mm: 21.4% and of 2 mm: 85.7%; RMS: 2.0 +/− 0.7 mm; ME for LL: 1.7 +/− 0.9 mm; chin: 2.0 +/− 1.0 mm; DL model had higher accuracy
2021, Alcañiz [39]	10	CBCT **	Yes **	FEM	LFI, LFII, BSSO, USSO, Ch, BIMAX	Surface with error < 3 mm with coarse meshes: 92%, with fine meshes: 95%
2022, Lee [10]	10	CBCT **	Yes **^c^	ProPlan CMF—FDM	BIMAX	Entire face ME: 0.73 +/− 0.21 mm, for LL: 1.42 +/− 0.77 mm, for UL: 1.14 +/− 0.80 mm, for chin: 0.95 +/− 0.58 mm; error < 2 mm: 90.9%
2022, Gutiérrez [40]	10	CBCT **	Yes **	FEM	LFI, LFII, BSSO, USSO, Ch, BIMAX	All distances for both meshes and their mean distances significantly < 2 mm, except LL, RGo, and LGo; distances for all landmarks significantly < 3 mm, except for LL of the fine mesh
2022, Yamashita [17]	88	CBCT	No	Dolphin 3D v.11.95—sparse landmark-based algorithm	BIMAX, TRIMAX	C II: underprediction with downward direction in S-Y, S-Z, LI-Y, SB-Y, Pog-Y, Pog-Z, Gn-Y, Gn-Z, Me-Y, Me-Z, values > 2 mm: LI-Y, SB-Y, Pog-Y, Gn-Y, Gn-Z, Me-Y; MaxE LI-Y: 2.73 mm. C III: overprediction and downward direction in Pog-Z, Gn-Y, Gn-Z, Me-Y, and Me-Z, all discrepancies < 2 mm
2022, Ma [8]	40	MSCT **	No	FSC-Net, point cloud DL	NR	Qualitative: FSC-Net comparable with FEM-RLSE; quantitative: landmarks entire face ME: 2.95 +/− 0.61 mm; surface entire face ME: 1.55 +/− 0.30 mm, lips: 1.58 +/− 0.26 mm, chin: 2.11 +/− 0.77 mm; FSC-Net comparable with FEM-RLSE
2022, Awad [34]	20	CBCT	Yes	IPS CaseDesigner v.2.1.4.4—MTM	BIMAX	Entire face ME: −1.5 to 1.4 mm, UL: −2.5 to 1.3 mm, LL: −2.1 to 2.5 mm, chin: −1.8 to 2.6 mm
2022, Hou [31]	58	CBCT	Yes ^c^	ProPlan CMF—FDM	BIMAX	Entire face ME: 1.43 +/− 0.40 mm; error of UL, LL, chin, right external buccal, and left external buccal > 2.0 mm; LL the least predictable: 2.69 ± 1.25 mm
2023, Şenyürek [30]	16	CBCT **	No	ProPlan CMF v.3.0—FDM	LFI	Error in UL and LL: 1.49 +/− 0.77 mm, in cheeks: 0.98 +/− 0.34 mm, nose: 0.86 +/− 0.23 mm, and eyes: 0.76 +/− 0.32 mm
2023, Ruggiero [47]	5	CBCT + MRI	No	FEM with patient-specific model generated from CBCT and MRI	BIMAX	Midface ME: 0.55 +/− 2.29 mm
2024, Fang [48]	40	MSCT **	No	DL, ACMT-Net with the CPSA module	BIMAX	Quantitative: surface entire face ME: 1.06 +/− 0.43 mm, UL: 1.13 +/− 0.71 mm, LL: 1.23 +/− 0.48 mm, chin: 1.13 +/− 0.62 mm; landmarks entire face: ME 2.44 +/− 0.45 mm, upper face: 1.23 +/− 0.47 mm, lower face: 3.25 +/− 0.66 mm Qualitative: 77.5% (31/40) of the simulations clinically acceptable

* Not clear; ** device not specified; ^a^ details in Table S4; ^b^ most relevant results; ^c^ 3D photograph fused with the MSCT/CBCT skin surface; BIMAX, bimaxillary osteotomy; BSSO, bilateral mandibular sagittal split osteotomy; Ch, genioplasty; ChL, cheilion left; ChR, cheilion right; ComL, left commissure; ComR, right commissure; DL, deep learning; FDM, finite difference method; FEM, finite element model; FNA, frontonasal angle; Gn, soft tissue gnathion; IR, infraorbital rim; IQLFIIO, intraoral quadrangular Le Fort II osteotomy; lat ceph, lateral cephalograms; LFI, Le Fort I maxillary osteotomy; LGo, soft tissue left gonion; Li, crown of the lateral incisor; LI, labrale inferior; LL, lower lip; LS, labrale superior; MaxE, maximal error; Me, soft tissue menton; ME, mean error; MinE, minimal error; MSM, mass spring model; MTM, mass tensor model; NLA, nasolabial angle; NR, not reported; PFEM, probabilistic FEM; Pog, soft tissue pogonion; RGo, soft tissue right gonion; RMS, root mean square; S, stomion; SA, soft tissue A point; SB, soft tissue B point; SF, sinus floor; SN, subnasale; ST, soft tissue; TRIMAX, bimaxillary osteotomy and genioplasty; UL, upper lip; USSO, unilateral mandibular sagittal split osteotomy.

In the majority of studies, real bony movements were used for the generation of 3D soft tissue simulations; however, in nine studies [8,17,25,26,27,32,34,43,46], measurements were only based on the initial virtual treatment plan.

In the studies reviewed in this SR, the following two quantitative analysis methods were used: (1) 3D landmark-based evaluation was performed in 18 studies [8,11,14,15,17,18,21,22,23,25,26,27,31,32,38,40,45,48], and (2) surface mesh-based evaluation was performed in 28 studies [3,8,9,10,16,19,20,22,23,24,28,29,30,31,33,34,35,36,37,39,41,42,43,44,45,46,47,48]. In six studies [8,22,23,31,45,48], both methods were combined. 

Furthermore, the definition of accuracy, referring to the comparison of the actual postoperative soft tissue outcome to the 3D soft tissue simulation, varied between the studies reviewed in this SR. Some studies defined accuracy as a clinically insignificant error of less than 0.5 mm, while others used thresholds of 1 mm, 2 mm, or even up to 3 mm.

Regarding the mean error of the 3D soft tissue simulations of the whole face, fluctuations from 0.27 mm [38] to 2.9 mm [8,15] were reported. Due to variability in analysis methods, however, direct comparison is not possible. In the studies evaluating 3D soft tissue simulation accuracy after a Le Fort I osteotomy only, the upper lip and paranasal regions were reported to have the largest error [15,19,30,37,45], while after an isolated bilateral sagittal split osteotomy (BSSO), the largest error was reported for the lower lip and chin regions [22,33]. In the studies evaluating simulation after bimaxillary osteotomy with or without genioplasty, the highest inaccuracy was reported at the level of the lips, predominantly the lower lip, chin, and, sometimes, the paranasal regions [9,10,14,17,23,27,29,31,32,34,36,43]. 

The overall inconsistency in methodology encouraged the authors of this SR to summarize the various methodologies (shown in Table 5), since such inconsistency could be considered an additional risk of bias. 

## 4. Discussion

Three-dimensional VTP has become the state of the art for surgical planning for patients needing orthognathic surgery. Nonetheless, there is still a significant lack of evidence-based data regarding the accuracy of its associated 3D soft tissue simulation. In order to discuss the findings of this SR in a structured manner, a framework was set up, which resulted in a proposal of guidelines (Table 6) to systematize the workflow for evaluating the accuracy of 3D soft tissue simulations in orthognathic surgery in future studies.

### 4.1. Image Acquisition (Pre- and Postoperative)

In the studies reviewed in this SR, different medical image acquisition techniques were reported: MSCT, CBCT, and 3D photographs and MRI. These were used separately or in combination. Only a few studies fully reported their pre- and postoperative image acquisition protocol details: imaging device, patient’s head position, lip morphology and posture, mandible positioning (centric relation, centric occlusion, the use of wax bite), and time interval between the surgery and postoperative image acquisition [15,16,17,19,22,24,32,33,34,35,37,38,44]. Most of the studies only considered these details partially. Regarding the imaging device and patient’s head position, 14 studies reported an MSCT scanning protocol performed in a horizontal position. Only one study presented a scanning protocol with a CBCT apparatus that scanned the patient in a supine position [28]. This is crucial for clinicians, since scanning the patient in a horizontal position inherently modifies and falsifies the 3D facial soft tissue mask due to the effects of gravity [49]. A study by Iblher et al. [50] showed that gravitational facial soft tissue deformation could range from 4 to 6 mm when comparing horizontal and vertical image acquisition. Therefore, it is of paramount importance to scan the patient in a vertical position, avoiding gravitational distortion of the facial soft tissues. An additional advantage of CBCT scanning compared to MSCT is that patients are exposed to a much lower radiation dose [51]. A limiting factor of CBCT scanning, however, is a potentially smaller field of view (FOV) in some CBCT devices, which can result in incomplete capture of the facial soft tissue mask, e.g., the tip of the nose [32]. Currently, the majority of CBCT scanners are equipped with specific algorithms to partly solve this issue. Unfortunately, the nose tip still cannot always be visualized with the correct 3D geometry. Stratemann et al. [52] observed statistical differences in measurements between different CBCT devices (NewTom and CB MercuRay). Therefore, to superimpose and compare two sets of CBCT data, it is important that the image acquisition is performed with the same CBCT device. 

The limitations of 3D soft tissue simulation that were identified in the studies included in this SR also relate to lip morphology and posture, which indicates the importance of a standardized scanning protocol. It has repeatedly been suggested [22,23,33,38,53] that it is important for patients to relax their lips to avoid muscle hyperfunction during scanning.

In eight studies [10,15,16,21,22,23,28,31], the generated 3D soft tissue simulations were obtained from preoperative images with fixed orthodontic appliances in place, while the postoperative image acquisition was performed after these appliances had been removed. Resnick et al. [15] and Liebregts et al. [23] indicated that this could probably have influenced the final lip position and morphology. Eidson et al. [53] and Kim et al. [54] used 3D stereophotogrammetry and reported statistically significant differences in the right and left commissures, as well as the lower lip, after the orthodontic appliances were removed.

In the presented studies, the time interval for postoperative CBCT image acquisition varies from 3 months [26,46] to 72 months [32]. In a prospective study by van der Vlis et al. [55], which quantified changes in postoperative swelling, it was reported that 50% of facial swelling resolves within the first three weeks post operation, 20% persists after three months, and 11.2% of the initial swelling volume remains at six months. Moreover, facial soft tissue swelling continues to decline at a statistically significant rate from six to twelve months postoperatively.

### 4.2. Virtual Osteotomies and VTP

The accuracy of 3D soft tissue simulation depends on two main factors: the computation model itself and the mismatch between surgical planning and the actual surgical movements [16]. Khambay and Ullah [45] and Baan et al. [56] stated that surgeons are generally unable to transfer the virtually predicted surgical plan perioperatively in an accurate way, and significant errors are introduced. Knoops et al. [16] compared the accuracy of 3D soft tissue simulation based on planned skeletal movements and actual postoperative bony movements. An increase in root mean square distance between the simulation and postoperative soft tissue outcome was observed when using the initially planned segments positions. Therefore, the analysis method should rely on determining the exact skeletal changes that occurred after the surgery (and any potential relapse). The use of the initial bony virtual surgical plan as a template to evaluate the accuracy of the 3D soft tissue simulation may cause a discrepancy between 3D soft tissue simulation and postoperative facial outcome and bias the accuracy of the results.

### 4.3. Considerations Regarding Additional Surgical Procedures

According to Holzinger et al. [35], the higher rate of error in predicting the 3D outcome of the soft tissues in the paranasal region and upper lip could be explained by additional intraoperative surgical maneuvers, such as septoplasty, reshaping of the anterior nasal spine (ANS) or nasal base, or soft tissue closure methods. Current 3D virtual planning software programs cannot reproduce the effect of different suturing techniques, resulting in non-linear soft-to-hard-tissue ratios, especially in the lip regions [32]. Thus, additional surgical techniques are, in fact, an uncontrolled factor that jeopardizes the 3D soft tissue simulation algorithm to a potentially clinically relevant extent. Nevertheless, Liebregts et al. [23] found a statistically significant favorable result for 3D simulation of the facial soft tissue mask when performing V-Y closure compared to surgeries without V-Y closure. With regard to the alar cinch suture, their findings were not significant. Moreover, additional surgical procedures were only briefly reported in 12 studies [15,16,17,18,19,20,21,23,27,32,37,38]. To improve the accuracy of 3D soft tissue simulation, additional surgical procedures, such as septoplasty, rhinoplasty, bony reshaping (ANS, nasal base, lateral nasal walls, chin, gonial angles, and zygomas), bone augmentation (grafts and PSIs), soft tissue closure methods after Le Fort I osteotomy (V-Y closure, alar base cinch suture, and paranasal cross sutures), lipofilling, or liposuction, should be reported in the methodology of the study and, ideally, in the future, they should be incorporated into the 3D soft tissue simulation model. Ter Horst et al. [33] therefore suggested a deep-learning-based algorithm as a suitable model for incorporating all these factors. 

### 4.4. Soft Tissue Simulation Algorithms

The simulation of a patient’s new facial outlook requires a mathematical model that can process the deformation of the facial tissues due to underlying bony movements [3]. The algorithms of computational modeling that have been applied to 3D facial soft tissue morphing can be divided into five broad categories: (1) mass spring models (MSMs), (2) finite element models (FEMs), (3) mass tensor models (MTMs), (4) sparse landmark-based algorithms, and (5) methods that use artificial intelligence (AI). Each of these have their particular advantages and drawbacks [57], which are presented in Table 7. Mollemans et al. [3] compared four different computational strategies: a linear FEM, a non-linear FEM, an MSM, and an MTM. They found that the most accurate results were obtained with the FEM and the MTM.

No mathematical model, however, has been generally accepted as the gold standard [15]. 

Recently, AI applications have spread rapidly in various fields of medicine and maxillofacial surgery [60]. The rationale for incorporating AI in soft tissue simulation methods is that it improves accuracy. AI includes machine learning (ML), which comprises both deep learning (DL) and artificial neural networks (ANNs) [61]. In this SR, only four studies presented models based on DL [8,33,36,48], and the results showed that the 3D soft tissue simulation accuracy is comparable to [8] or surpasses [33] the accuracy of the mathematical biomechanical algorithms. AI models require a huge database of MSCT, CBCT, or MR images, including data on additional surgical procedures, which are currently not available [59]. Hence, Ter Horst et al. [33] suggested that a web-based data sharing platform, to which multiple centers can upload standardized preoperative, planned, and postoperative 3D data, is the most likely way forward.

### 4.5. Rigid Registration of Preoperative/Simulated and Postoperative Data

Superimposition of 3D data, also called image rigid registration or image fusion, involves the spatial alignment of similar structures (e.g., a CBCT soft tissue mask and a 3D photograph or a 3D virtual treatment plan and post-treatment imaging data) [49]. There are different types of rigid registration: landmark-based, surface-based, and voxel-based rigid registration [7]. In order to evaluate 3D soft tissue simulation and enable measurements, 4 studies used landmark-based [15,30,34,35], 17 studies [9,10,16,18,19,28,29,31,32,33,37,38,41,43,44,45,47] applied surface-based, and 10 studies [3,14,17,20,21,22,23,24,25,29] used voxel-based rigid registration of the simulation and actual outcome. Voxel-based registration processes the raw information of a DICOM image by using the gray scale intensity of the voxels for superimposition. In contrast, surface-based registration requires an additional step of 3D model rendering to generate a 3D surface mesh model, which leads to a potential source of error [62]. Point-based rigid registration only uses corresponding points to compute the rotation and translation between datasets [49] and is prone to human error due to manual tracing of 3D cephalometric landmarks. Moreover, it has been shown to be inferior to surface- and voxel-based registration [63], which was confirmed by Andriola et al. [64], who showed in an SR that voxel-based superimposition protocols presented the highest accuracy and reproducibility. Voxel-based registration should, however, ideally be performed using only one type of user-independent software and based on a stable volume of interest (VOI) (e.g., the anterior cranial base, the total cranial base, or both zygomatic arches).

On the other hand, 3D photographs, as a non-ionizing imaging method, have relevant clinical potential for diagnosis and longitudinal non-radiation virtual treatment outcome analysis [49]. A total of eight studies in this review used a fusion of MSCT or CBCT data with 3D photogrammetry images to replace the MSCT or CBCT 3D soft tissue mask. Resnick et al. [15] stated that errors are created with each additional step in the imaging registration process, and thus, inferior accuracy can be expected with subsequent registration of a 3D photograph. Image registration errors were mainly located in the cheek and orbital regions and were reported to be larger than 1.5 mm [65].

### 4.6. Postprocessing and Analysis

The evaluation of the accuracy of 3D soft tissue simulation may be either quantitative or qualitative. 

Quantitative validation measures the error between the virtually simulated 3D orthognathic treatment plan and its actual postoperative results. Qualitative validation uses questionnaires that are answered by surgeons or shape analysis [39]. In the reviewed studies, two main quantitative analysis methods have been used: (1) 3D landmark-based evaluation, where linear and angular differences between reference points placed on superimposed predicted and postoperative 3D models are measured, and (2) surface mesh-based evaluations, where surface-to-surface distances are measured [10]. This SR showed that 3D landmark-based evaluation has important shortcomings. One of them is the variability in the identification of 3D cephalometric landmarks [40], which is prone to human error, ranging from 0.3 to 2.8 mm, particularly when the landmarks have to be placed manually [7,66]. This source of error, however, could be minimized by the automatic identification of landmarks, for example, by means of AI algorithms [40]. In this SR, 7 [8,17,21,22,25,27,32] out of the 18 studies using landmark evaluation methods reported employing corresponding points, while others did not report correspondences or used the distance between one point on one surface and the closest point on the second surface. This might not be the corresponding anatomical point and would result in an underestimation of the error [45]. In the surface mesh-based method, there is no need to define 3D cephalometric landmarks, which eliminates the potential errors associated with this process. However, in the majority of the studies in this SR, measurements were taken using the minimal Euclidean distance between the two nearest points of the two surface meshes (the shortest distance between the triangle vertices of the adjacent meshes), with no actual anatomical correspondence [67]. This may explain the resulting underestimations of the error. Therefore, in a few studies [3,20,35,36], the authors used a generic mesh to overcome this problem. The generic mesh is a 3D virtual mask that resembles the human face, with a predefined number of equally sized triangles and a set of indexed vertices. It has the potential to mimic the morphology of a specific face by creating a deformation through a process known as “conformation”. Conformation enables the vertices that have been displaced by morphological changes (e.g., simulation or surgery) to be tracked. This provides an anatomical correspondence of vertices in two surface meshes [68,69]. Many studies have assessed the error of 3D soft tissue simulation in regard to the entire face, which includes large areas that are not affected at all by the performed surgery and could decrease the actual error [10]. Furthermore, when reporting based on the entire face, the site of the error remains unknown and is not clinically meaningful. In the studies that divided the face, the anatomical regions involved in orthognathic surgery were defined by the authors themselves [31]. 

Kim et al. [9], however, suggested surface deviation error alone to be an intuitive notion rather than a representation of the true anatomical correspondence. The unnatural shape and position of the lips, the labio-mental fold, the chin, and the soft tissue mesh distortion in the cheek regions next to the inferior border of the mandible could only be recognized by qualitative analysis using the “clinical human eye”. Therefore, in addition to quantitative analysis, they introduced a qualitative evaluation and confirmed that the quantitative error does not necessarily correspond to the clinicians’ qualitative evaluation. This was their rationale for introducing a lip shape analysis [41], evaluating the geometrical difference in the 2D lip profiles between the soft tissue simulation and the postoperative outcome. 

Shafi et al. [19] reported that the overpredicted displacement of the soft tissue in the 3D simulation (positive values) and the underpredicted displacement (negative values) should be treated equally, i.e., as absolute values, to measure the mean difference. If some parts of the simulated surface lie behind and some in front of the postoperative surface, distance measurements for this region would comprise signed distances, i.e., positive and negative values. Any positive values would cancel out any negative values, thus underestimating the mean error and thereby biasing the results [45]. Therefore, the absolute mean values or Euclidean distances and the root mean square error should be measured.

### 4.7. Accuracy Cut-Off

Finally, in order to compare results reported in the literature regarding the accuracy of 3D soft tissue simulation in orthognathic surgery, it is important to define what accuracy means. In the studies reviewed in this SR, accuracy, referring to the comparison of the actual postoperative soft tissue outcome to the 3D soft tissue simulation, was defined in different ways [40], as an error of less than 0.5 mm, 1 mm, 2 mm, or up to 3 mm. Lee et al. [10] suggested setting the value of clinical insignificance at 2 mm, as this has been proposed in conventional 2D lateral cephalometric analysis. Kim et al. [9] reported a clinically acceptable error between the simulated and the actual soft tissue result below 2 mm (mean error) or 3 mm (maximum error). However, while there have been different attempts to set a fixed value of error that is clinically acceptable (i.e., unnoticeable by a lay person’s eye), there is no consensus in the current literature. Further studies with a proper study design are necessary in the future in order to gain evidence-based data.

## 5. Conclusions

This systematic review aimed to evaluate the accuracy of 3D soft tissue simulations in orthognathic surgery that have been reported in the literature. The findings underscore the diverse methodologies and approaches used in assessing simulation accuracy, emphasizing the critical need for standardization in this domain. 

The current software packages and algorithms used in 3D soft tissue simulations have inherent limitations. A mean error varying from 0.27 mm to 2.9 mm for 3D soft tissue simulations for the whole face was reported. In the studies evaluating 3D soft tissue simulation accuracy after a Le Fort I osteotomy only, the upper lip and paranasal regions were reported to have the largest error, while after an isolated bilateral sagittal split osteotomy, the largest error was reported for the lower lip and chin regions. In the studies evaluating simulation after bimaxillary osteotomy with or without genioplasty, the highest inaccuracy was reported at the level of the lips, predominantly the lower lip, chin, and, sometimes, the paranasal regions.

The integration of artificial intelligence (AI) algorithms represents a promising advancement in enhancing the accuracy and efficiency of 3D soft tissue simulation. However, further research and a huge database of MSCT, CBCT, or MR images are needed to fully leverage the potential of AI in this context.

Due to significant variability in methodology and study design, meta-analysis was not feasible. Therefore, based on the results of this SR, guidelines to systematize the workflow for evaluating the accuracy of 3D soft tissue simulations in orthognathic surgery are proposed. These guidelines aim to streamline future research efforts and enhance comparability across studies.

In conclusion, while 3D soft tissue simulation holds promise for improving surgical outcomes in orthognathic procedures, ongoing efforts to address methodological challenges and advance technology are essential to realize its full potential in clinical practice.

## Figures and Tables

**Table 1:** PICOS format.

Systematic Search Strategy/PICOS Format	
(P) Population	Patients with class I, class II, class III, or asymmetric dentoskeletal deformities who underwent orthognathic surgery (Le Fort I, II osteotomy, bilateral sagittal split osteotomy, bimaxillary osteotomy, genioplasty)
(I) Intervention	Three-dimensional soft tissue simulation in VTP
(C) Comparison	Comparison of different methods or approaches for assessing the accuracy of 3D soft tissue simulation. Comparison of various software platforms or algorithms utilized for 3D soft tissue simulation
(O) Outcome	Accuracy of 3D soft tissue simulation in VTP
(S) Study design	Pro- and retrospective studies with a minimum sample size of 3 subjects

VTP, virtual treatment planning.

**Table 2:** Inclusion and exclusion criteria.

Inclusion Criteria	Exclusion Criteria
Studies that used VTP and 3D soft tissue simulation for orthognathic surgery planning Comparison between 3D soft tissue simulation and postoperative soft tissue outcome for 3D soft tissue simulation Postoperative record acquisition at least 3 months after surgery Papers in English, Dutch, German, or Polish	Syndromic and cleft patients Case reports, studies with <3 study subjects

**Table 3:** Reported software packages used for VTP and 3D soft tissue simulation.

Software		References
Dolphin 3D software	(Dolphin Imaging & Management Solutions, Chatsworth, CA, USA)	[14,15,16,17,18]
Maxilim	(Medicim NV, Mechelen, Belgium)	[11,19,20,21,22,23,24,25]
SimPlant ProOMS	(Materialise, Leuven, Belgium)	[26,27]
SurgiCase CMF Pro	(Materialise, Leuven, Belgium)	[28,29]
ProPlan CMF	(Dentsply-Sirona, York, PA, USA; Materialise, Leuven, Belgium)	[10,16,30,31]
OrtogOnBlender-OOB	(Blender Foundation)	[32]
IPS Case Designer	(KLS Martin Group, Tuttlingen, Germany)	[18,33,34]
SOTIRIOS software	(University of Basel, Switzerland)	[35]

**Table 5:** Methodological data on the studies included in this review.

Year, Author	Image Acquisition				Real Bony Movement	Additional Procedures	Type of Rigid Registration Method for Soft Tissue Evaluation (VOI for Superimposition)	Method of Analysis		Fixed Point of Accuracy
	Patient Position	Lip Position	CR/Wax Bite/CO	Postop Imaging Time Interval			a. Landmark-Based, LB; b. Surface-Based, SB; c. Voxel-Based, VB; d. Registration-Free Method, RF	Landmarks	Surface to Surface	
2004, Chabanas [42]	H	NR	NR	NR	Yes	NR	*		Entire face; closest point; signed Euclidian distances	NR
2007, Mollemans [3]	H	NR	NR	4 mos	Yes	NR	VB (top of skull)		Entire face—region of interest; corresponding points; signed Euclidean distances; mean, variance, 50%, 90%, and 95% percentiles of distance distributions; qualitative validation: surgeons’ visual inspection	NR
2007, Marchetti [46]	H	NR	NR	3–6 mos	No	NR	VB *		Face surface—region of interest; mean distance, % of simulations with error < 2 mm	2 mm
2010, Bianchi [28]	H	NR	NR	6 mos ^a^	Yes	NR	SB (soft tissue—forehead and eyes)		Entire face; closest point; average absolute error, SD and max, and 90th and 95th percentiles; % with error equal to or < 2 mm	2 mm
2010, Ulusoy [43]	H	NR	NR	NR	No	NR	SB		Entire face; closest point; mean differences	NR
2011, Centenero [26]	H/V	NR	CR, wax bite	3 mos	No	NR	RF	Landmarks; difference between linear and angular measurements within each face; ICC between measurements (“high”, “medium”, and “low” correlation)		NR
2011, [29] Marchetti	H	NR	NR	6 mos	Yes	NR	SB (soft tissue—forehead and eyes)		Entire face; closest point; mean absolute error, SD, max, and 90th and 95th percentile; % of simulations with error equal to or <2 mm	2 mm
2013, Schendel [38]	V	Relaxed	CR *	6 mos	Yes	Reconstruction of nasolabial muscles *	SB *	Eighteen landmarks (10 midline, 8 lateral); signed mean values, absolute mean values, SD, RMS difference for all measurements		0.5 mm
2013, Shafi [19]	V	Relaxed	CO, wax bite	6–12 mos	Yes	ANS plasty; alar cinch suture; V–Y closure	SB (soft tissue—forehead)		Eight regions; mean absolute error, SD, 95% CI	3 mm
2013, Nadjmi [11]	NR	NR	CR, wax bite	4 mos	Yes	NR	Two-dimensional best fit and superimposition of SNL and OCSNL	Fifteen midline landmarks; differences across *x*-axis and *y*-axis: signed mean, SD, min, max, and frequency of clinically acceptable error (%) +/− 0.5 mm; nasolabial and mentolabial angles		0.5 mm
2014, Terzic [44]	H/V	Relaxed	CO *	6 mos	Yes	NR	SB *		Upper and lower half of face (pupil line), closest point, signed mean difference, SD, % of mass spring points with error < ±1 mm and exceeding ±1, ±2, and ±3 mm	1 mm
2014, Nadjmi [24]	V	Relaxed	CR, wax bite	4 mos	Yes	NR	VB (between infraorbital rim and rest of viscerocranium)		Entire face; closest point; mean absolute difference; mean signed distance, 25–75% distance range, 5–95% distance range	2 mm
2015, Ullah [37]	V	Relaxed	CO; wax bite	6–12 mos	Yes	ANS plasty; alar cinch suture; V–Y closure	SB (anterior cranial base, skull vault)		Eight regions; 90th percentile mean absolute error, SD, 95% CI	3 mm
2015, Khambay [45]	NR	NR	NR	Min 6 mos	Yes	NR	SB (skull base)	Ten landmarks (six midline; four lateral); closest distance between two surface meshes at that point; arithmetic mean, absolute mean; SD	Entire face and 8 regions; closest point; max and absolute mean, 95th and 90th percentiles; SD; % of 3D points equal to or <2 mm; RMS error	2 mm
2015, Nam [27]	H	NR	NR	6 mos	No	Alar cinch suture	*	Ten landmarks (six midline, four lateral); corresponding; means, SD; absolute values and vector values using x, y, and z coordinates; accuracy rate: <2 mm		2 mm
2015a, Liebregts [23]	V—seated	Relaxed	NR	Min 6 mos ^a^	Yes	Alar cinch suture; V-Y closure	VB (cranial base, forehead, zygomatic arches)	Six midline landmarks; corresponding; Euclidean distances; mean absolute error; SD; max and min absolute error; 95% CI	Entire face and 3 regions; closest point, mean absolute error, SD, range and 95th percentile; % of simulations with error equal to or <1 mm and 2 mm	2 mm
2015b, Liebregts [22]	V—seated	Relaxed	CR, wax bite	Min 6 mos ^a^	Yes	NR	VB (cranial base, forehead, zygomatic arches)	Six midline landmarks; corresponding; Euclidean distances; mean absolute difference, SD	Entire face and 3 regions; closest point, mean absolute error, SD, 90th percentile, % of simulations with error equal to or <1 mm and 2 mm	2 mm
2015, Van Hemelen [25]	V—seated	NR	CR, wax bite	4 mos	No	NR	VB (between infraorbital rim and rest of viscerocranium)	Nine landmarks in midsagittal plane; corresponding; difference in depth (Y), in height (Z), and 2D distance in sagittal plane (NR)		2 mm
2016, Liebregts [21]	V—seated	Relaxed	NR	Min 6 mos^a^	Yes	ANS plasty, nasal base plasty, alar cinch suture; V-Y closure	VB (cranial base, forehead, zygomatic arches)	Three landmarks; corresponding; Euclidean distances; mean absolute error, SD, range		NR
2016, Resnick [15]	V	Relaxed	CO	Min 6 mos ^a^	Yes	Alar cinch suture	LB *	Fourteen landmarks * (six midline, eight lateral) and nasolabial angle; mean error, % of average absolute error <2 mm		2 mm
2017, Kim [9]	H	NR	NR	MSCT 6 weeks/3D photo min 6 mos	Yes	NR	SB (forehead and nasal bridge)		Entire face and 8 subregions; mean errors, SD, and max errors (absolute mean Euclidean distances along normal vectors); clinicians’ qualitative evaluation: binary visual scoring scale (Unacceptable; Acceptable)	Mean: 1.5 mm; max: 3 mm
2021, Kim [41]	H	NR	NR	MSCT 6 weeks/3D photo min 6 mos	Yes	NR	SB (forehead and nasal bridge)		Entire face and 6 regions; mean error (absolute Euclidean distances along surface normal vectors); qualitative evaluation: lip shape analysis	NR
2017, Mundluru [20]	V	NR	NR	6–12 mos	Yes	Alar cinch suture; V–Y closure; condylectomy	VB (skull base)		Eight anatomical regions; min, max, mean, SD, absolute max, absolute mean, and absolute SD of 90% of points; directional discrepancies at each vertex in x, y, and z dimensions separately—conformed generic meshes—corresponding points	2 mm
2018, Holzinger [35]	H	Relaxed	CR	6 mos	Yes	NR	LB		Entire face; corresponding points; mean error, SD, median, 80th, 95th, 99th, and 99.9th percentiles	2 mm
2019, Knoops [16]	V	Relaxed	CO	12 mos ^a^	Yes	Alar cinch suture	SB (skull base)		Midface: upper lip and paranasal regions; closest point; RMS distance; % of points <2 mm	2 mm
2019, Elshebiny [14]	V	NR	CO	6–12 mos	Yes	NR	VB (cranial base)	Landmarks; difference between 12 linear and 3 angular measurements within each face; means, SD		1.5 mm
2021, Cunha [32]	H	Relaxed	CR, wax bite	Min 6 mos	No	Alar cinch sutures, V-Y closure	SB *(skull base, nasal bones, frontal bone, zygomatic arches)	Seventeen landmarks (five midline, twelve lateral); corresponding; Euclidean distance; mean deviation, 95% CI, max, min, SD		2 mm
2021, Willinger [18]	NR	NR	NR	Min 4 mos	Yes	Camouflage of infraorbital step with milled bone and fibrin glue	SB* (maxilla)	Six landmarks; three lateral landmarks at both sides along MFAL—Midfacial Advancement Line technique; mean error, SD, median, 95% CI, variants, min, max, range, interquartile range, skewness, and kurtosis		2 mm
2021, Tanikawa [36]	V	NR	NR	NR	NR	NR	Common coordinate system based on landmarks		Entire face; corresponding points; differences in *z*-axis; average error, SD, min and max; % of cases with average error <1 mm or <2 mm	1 mm, 2 mm
2021, ter Horst [33]	V	Relaxed	CO	CBCT: 12 mos; 3D photo 5–19 mos	Yes	NR	SB * (soft tissue)		Three anatomical regions; closest point; mean absolute error, RMS error, SD, 95th percentile; % of simulations with a max error of 1 mm and 2 mm	1 mm, 2 mm
2021, Alcañiz [39]	NR	NR	NR	NR	Yes	NR	NR		Entire face; signed closest-point distance; cumulative surface % with error < 3 mm; comparison of fine and coarse meshes	3 mm
2022, Lee [10]	NR	NR	NR	Min 6 mos ^a^	Yes	No	SB * (forehead and nose bridge)		Entire face (with upper third removed), 6 anatomical regions; closest point; mean absolute difference, SD; % of 3D points with error equal to or <2 mm; absolute mean, RMS for 95th percentile	2 mm
2022, Gutiérrez [40]	NR	NR	NR	NR	Yes	NR	NR	Nine landmarks (seven midline, two lateral); closest point; 25th, median, 75th; surgeons’ qualitative evaluation: Likert scale and binary questions; comparison of fine and coarse meshes		2 mm, 3 mm
2022, Yamashita [17]	V—seated	Relaxed	CR, wax bite *	6–12 mos	No	Alar cinch suture, suspension of mentalis muscle	VB (cranial base)	Landmarks (midsagittal plane); corresponding, signed distances in coordinate y (anteroposterior direction) and z (superoinferior direction)		2 mm
2022, Ma [8]	H	NR	NR	NR	No	NR	NR	Landmarks; corresponding *, Euclidean distance; mean error, SD, max error	Entire face and 5 subregions; Chamfer distance and Hausdorff distance; mean error, SD, max error; clinicians’ qualitative evaluation	NR
2022, Awad [34]	V—seated	Relaxed	CR *	4 mos	No	No	LB		Entire face and 6 regions; absolute discrepancies, unsigned mean absolute discrepancies (RMS), SD, % of surface with error < 2 mm = % of surface congruence (IO%)	2 mm
2022, Hou [31]	NR	Relaxed	*	Min 6 mos ^a^	Yes	NR	SB * (soft tissue forehead, nose bridge)	Landmarks (8 midline, 6 lateral); corresponding; differences on x, y, and z planes	Entire face and 10 regions; RMS distance, SD, 95% CI	2 mm
2023, Şenyürek [30]	NR	NR	NR	11–15 mos	Yes	NR	LB		6 anatomical regions; mean error, SD	2 mm
2023, Ruggiero [47]	CBCT: V standing; MRI: H	NR	CO, wax cast	6 mos	Yes	NR	SB		Midface; the closest point; mean error, SD	NR
2024, Fang [48]	H	NR	NR	NR	Yes	NR	*	Twenty landmarks (eight upper face, twelve lower face); Euclidean distance, mean error, SD	Entire face and 6 regions; mean error (average surface deviation error, SD); qualitative evaluation: lip-shape analysis	NR

ANS, anterior nasal spine; CI, confidence interval; CO, centric occlusion; CR, centric relation; H, horizontal; ICC, intraclass correlation coefficient; max, maximum; min, minimum; mos, months; NR, not reported; OCSNL, outer canthus–soft tissue nasion line; RMS, root mean square; SD, standard deviation; SNL, sella nasion line; V, vertical; * not clear; ^a^ no orthodontic appliances in the postoperative images; %, percentage.

**Table 6:** Guidelines to systematize the workflow for evaluating the accuracy of 3D soft tissue simulation in orthognathic surgery in future studies, based on this SR.

Workflow	Guidelines
1. Image acquisition (pre- and postoperative)	Report the details of the CBCT apparatus and the pre- and postoperative image acquisition protocol; CBCT in a vertical position (seated or standing) without deformation of the facial soft tissue mask, with the mandible in “centric relation”; extended FOV; the same CBCT device pre- and postoperatively; postoperative CBCT at least at 6 months after surgery to evaluate soft tissue changes and skeletal relapse; fixed orthodontic appliances should be in place
2. Image data processing	
Three-dimensional rendering of DICOM, STL, and OBJ files	Perform and evaluate 3D soft tissue simulation of the CBCT facial soft tissues; avoid the superimposition of the 3D photograph for analysis
Virtual osteotomies and VTP	Determine the exact skeletal changes that occurred after the surgery and any potential relapse (postoperative long-term CBCT); use the postoperative hard tissues as a template for analysis
Additional surgical procedures	Report additional surgical procedures, such as septoplasty, rhinoplasty, bony reshaping (ANS, nasal base, lateral nasal walls, chin, gonial angles, zygomas), bone augmentation (grafts, PSIs), soft tissue closure method after Le Fort I (V-Y closure, alar base cinch suture, paranasal cross sutures), lipofilling, and/or liposuction
3. Three-dimensional soft tissue simulation	Report the 3D soft tissue algorithm used for simulation
4. Rigid registration of preoperative/simulated data and postoperative data	Use voxel-based superimposition protocol using only one software, fully automated (user-independent) on a stable VOI (e.g., anterior cranial base, total cranial base, both zygomatic arches); report the software that was used
5. Postprocessing and analysis	
Quantitative analysis	Report absolute mean values or Euclidean distances and root mean square error
Three-dimensional landmark-based analysis	Use reliable and accurately definable corresponding points; automatic 3D landmark identification is recommended
Surface mesh-based analysis	Recommended true correspondences: generic/conformable mesh; only analyze facial surface that is affected by surgery; division of face into anatomical regions and separate analysis
Qualitative analysis	Objective evaluation method in addition to quantitative analysis is recommended

**Table 7:** Characteristics of 3D soft tissue simulation algorithms.

Algorithm	Characteristics	Software
Sparse landmark-based algorithm	→ Based on manually placed landmarks and interpolation between them [15,33] → Hard-to-soft-tissue ratios → Potential of hard-to-soft-tissue ratio adjustments for interpatient variability [16] → Larger errors compared to biomechanical modeling methods [15,16,18]	Dolphin^®^
Mass spring model (MSM)	→ Volumetric model [33] → Collection of point masses connected by linear or non-linear springs [29,32,38] → No real biomechanical foundation [57] → Low computational cost [29]	OrtogOnBlender^®^ 3dMDVultus^®^
Mass tensor model (MTM)	→ Volumetric model [33] → Biomechanical model in which tissue properties (elasticity, stiffness) are incorporated based on measurements within a clinical control group [21] → Strong biomechanical relevance [3] → Short computational time [3]	Maxilim^®^ IPS Case Designer^®^
Finite element model (FEM)	→ Volumetric model [33] → Creation of a high-quality patient-specific mesh and establishment of biomechanical properties and boundary conditions to mimic tissue behavior [8,58] → Possible implementation of detailed and patient-specific mesh for lips and realistic sliding effect between upper and lower lip and mucosa [41] → Strong biomechanical relevance [8] → Laborious data preparation and large internal memory usage, resulting in longer simulation times [3,8,47] → Real-time simulation is not possible [22,58]	
Artificial intelligence (AI)	→ Data-driven modeling [57] → Potential for incorporating patient-related factors (age, gender, soft tissue quality) and surgery-related factors (magnitude of bone movements, additional surgical procedures) [33] → Comparable to or surpasses accuracy of mathematical biomechanical algorithms [8,33] → Reduced time of data preparation and simulation [8,59] → Lower efficiency for rare facial deformities or large bone displacements and asymmetries [8,33] → Requires a huge database of MSCT, CBCT, or MR images—currently not available [59]	

## Supplementary Materials

The following supporting information can be downloaded at https://www.mdpi.com/article/10.3390/jimaging10050119/s1: Table S1: The full search string for each database; Table S2: Methodological data on the studies included in this review; Table S3: The revised Quadas2 tool for risk of bias and applicability assessment; Table S4: Image acquisition technique and device.
