# Supplementary material for: The Accuracy of Three-Dimensional Soft Tissue Simulation in Orthognathic Surgery—A Systematic Review
Source: J Imaging. 2024 May 14;10(5):119. doi: 10.3390/jimaging10050119 (PMC11122049; doi:10.3390/jimaging10050119)
Supplement: Supplementary file 1 [file jimaging-10-00119-s001.zip › jimaging-2965478-supplementary.pdf]

Table S1. The full search string of each database.

Source	Search terms
PubMed	*Concept 1: soft tissue*<br>Soft-tissue*[tiab] OR "face profile"[tiab] OR "facial profile"[tiab] OR profile-change*[tiab]<br>*Concept 2: orthognathic surgery*<br>"Orthognathic Surgery"[Mesh] OR orthognathic-surg*[tiab] OR jaw-surg*[tiab] OR "Orthognathic surgical procedures"[Mesh] OR maxillomandibular*[tiab] OR bimaxillary-surg*[tiab] OR "Mandibular advancement"[Mesh] OR mandibular-advance*[tiab] OR "mandibular setback"[tiab] OR "Osteotomy, Le Fort"[Mesh] OR Le-Fort*[tiab] OR LeFort*[tiab] OR "Osteotomy, sagittal split ramus"[Mesh] OR sagittal-split*[tiab] OR BSSO[tiab] OR SSRO[tiab] OR IVRO[tiab]
Embase	*Concept 1: soft tissue*<br>'soft tissue'/exp OR 'soft tissue*':ti,ab,kw OR 'face profile'/exp OR 'facial profile':ti,ab,kw OR 'profile change*':ti,ab,kw<br>*Concept 2: orthognathic surgery*<br>'orthognathic surgery'/exp OR 'orthognathic surg*':ti,ab,kw OR 'bimaxillary surg*':ti,ab,kw OR 'maxillo-mandibular*':ti,ab,kw OR 'jaw surg*':ti,ab,kw OR 'Maxillomandibular*':ti,ab,kw OR 'mandibular osteotomy'/exp OR 'mandibular advance*':ti,ab,kw OR 'mandibular setback*':ti,ab,kw OR 'sagittal split*':ti,ab,kw OR 'BSSO':ti,ab,kw OR 'SSRO':ti,ab,kw OR 'IVRO':ti,ab,kw OR 'Maxilla osteotomy'/exp OR 'Le Fort*':ti,ab,kw OR 'LeFort*':ti,ab,kw
Web of science	Concept 1: soft tissue<br>"Soft tissue*" OR "face profile" OR "facial profile" OR "profile change*"<br>Concept 2: orthognathic surgery<br>"Orthognathic Surg*" OR "jaw surg*" OR maxillomandibular* OR "bimaxillary surg*" OR "mandibular advance*" OR "mandibular setback" OR "Le Fort*" OR LeFort* OR "sagittal split*" OR BSSO OR SSRO OR IVRO
Cochrane	*Concept 1: soft tissue*<br>((Soft NEXT tissue*) OR face profile OR facial profile OR (profile NEXT change*)):ti,ab,kw<br>*Concept 2: orthognathic surgery*<br>([mh "Orthognathic Surgery"] OR [mh "orthognathic surgical procedures"] OR [mh "mandibular advancement"] OR [mh "osteotomy, Le Fort"] OR [mh "Osteotomy, sagittal split ramus"]) OR (orthognathic NEXT surg*)OR (jaw NEXT surg*) OR maxillomandibular* OR (bimaxillary NEXT surg*) OR (mandibular NEXT advance*) OR mandibular setback OR (Le NEXT Fort*) OR LeFort* OR (sagittal NEXT split*) OR BSSO OR SSRO OR IVRO):ti,ab,kw

Table S2. Methodological data of the studies included in the review.

Year, first author	Study design	Sample size	Mean age (years)	Gender	Diagnosis	CBCT/ MSCT[a]	3D photo[a]	Software package and/or algorithm	Type of surgery	Results[b]
2004, Chabanas	NR	3	NR	NR	NR	MSCT**	No	FEM	NR	ME range 1 - 1.5 mm, MaxE 3–6 mm
2007, Mollemans	RS	10	NR	NR	C II, C III	MSCT**	Yes	(1)linear FEM; (2)non-linear FEM; (3)MSM; (4)MTM	TRIMAX, BIMAX, BSSO, BSSO+Ch, LFI+Ch	The average median distance for MTM: 0.60 mm, FEM: 0.60 mm, MSM: 0.64 mm, NFEM: 0.63 mm, average 90th percentile distance for MTM: 1.48mm, FEM: 1.51mm, MSM: 1.67mm, NFEM: 1.71mm; Highest accuracy: FEM and MTM
2007, Marchetti	NR	25	25.1^^	F: 17, M: 8	NR	MSCT	No	VISU system	LFI, BSSO, LFI+Ch, BSSO+Ch, BIMAX, TRIMAX	Error < 2mm in 80% (20 of 25) of the patients
2010, Bianchi	NR	10	24	F: 4, M: 6	NR	CBCT	No	SurgiCase CMF Pro v.1.2	BIMAX/ TRIMAX	ME: 0.94 mm; error < 2 mm in 86.8% of the simulations; 90th percentile: 2.24mm, 95th percentile: 2.81 mm.
2010, Ulusoy	NR	6	24 ± 6.1	F: 2, M: 5*	*	MSCT**	No	Dynamic volume spline	BIMAX *	ME: 1.8 mm
2011, Centenero	PS	16	NR	NR	C II, C III, As	MSCT/ CBCT	No	SimPlant ProOMS v.10.1	BIMAX, TRIMAX, BSSO+Ch	5 of 8 ST measurements: high degree of correlation, 3 measurements: medium degree of correlation

2011, Marchetti	NR	10	NR	F: 5, M: 5	MH and mandibular prognathism	MSCT	No	SurgiCase CMF Pro v.1.2	BIMAX, TRIMAX	ME: 0.75 +/- 0.78 mm; error < 2 mm in 91% of the simulations; 90th percentile: 1.94 mm, 95th percentile: 2.47 mm
2013, Schendel	NR	23	31	F: 13, M: 10	Maxillary, mandibular retrusion, microgenia	CBCT	Yes[c]	3dMDVultus - MSM	LFI, BSSO, Ch	Entire face ME: 0.27 mm, ComR: 1.10mm, ComL 0.99mm, Pog 0.79mm
2013, Shafi	RS	13	23 ± 8	F: 8, M: 5	C III	CBCT	No	Maxilim v.2.2.0 - MTM	LFI	ME: 0.97 mm; all anatomical regions with error significantly < 3.0 mm, exception UL error: 2.73 +/- 1.72; overprediction of UL
2013, Nadjmi	NR	13	NR	F: 11, M: 2	NR	CBCT**, lat ceph	Yes**	(1)2D Dolphin v.10 - fixed hard-tissue to soft-tissue ratios; (2)Maxilim - MTM	LFI, LFI+Ch, BIMAX, TRIMAX	Dolphin range of error in horizontal position: -1.41 to 1.20 mm, in vertical position: -1.85 to 1.55 mm; Maxilim range of error in horizontal position: -1.60 to 1.50 mm, in vertical position: -4.25 to 2.42 mm. No statistical differences between softwares, exception SA in Maxilim
2014, Terzic	RS	13	25.2	F: 8, M: 5	NR	MSCT/ CBCT	Yes[c]	3dMDvultus v.2.2.0.8 - MSM	BSSO, BSSO+Ch, BIMAX, TRIMAX	ME for the upper part +0.27 mm, the lower part –0.64 mm; in the lower part error < +/-1mm 26.9%, > +/- 1mm 73,1%, > +/-2mm 49.5% and > +/-3mm 29.8%
2014, Nadjmi	NR	20	23 ± 9^^	F: 15, M: 5	C I, C II, C III, As	CBCT	No	Maxilim - MTM	BSSO, BIMAX, TRIMAX	ME: 1.18 mm; 84% of errors between -2 mm and +2 mm
2015, Ullah	RS	13	23 ± 8	F: 8, M: 5	C III	CBCT	No	3dMDVultus v.2.2.0 - MSM	LFI	ME: 0.92 mm (0.3–2.4 mm); 90th percentile from 0.65 mm (chin) to 1.17 mm (UL). ME significantly < 3 mm. The 95% CI in all regions < 2 mm
2015, Khambay	RS	10	NR	NR	NR	CBCT**	No	3dMDvultus v.2.2.0 - MSM	LFI	ME for 95th percentile: 0.98 mm - 0.56 mm, for 90th percentile: 0.91 mm - 0.50 mm; error < 2 mm: 94.4% - 85.2% points. The RMS error: 2.49 mm- 0.94 mm. The RMS difference for all measurements: 1.3 mm
2015, Nam	RS	29	NR	F: 13, M: 16	NR	MSCT	No	Simplant Pro	BIMAX, TRIMAX	ME in all landmarks: 2.03mm. Error < 2mm: 52.8%. Absolute error values in the x-axis: 0.73mm, y-axis: 1.39mm, z-axis 0.85mm; error significantly > 2 mm: ChR, ChL, LL, Pog. MaxE: 2.38mm in ChL, MinE: 0.84mm in pronasale.
2015a, Liebregts	RS	60	26	F: 45, M: 15	NR	CBCT	No	Maxilim - MTM	BIMAX	Landmarks: MaxE at LI 3.1 +/- 1.4 mm, MinE at SN 1.5 +/- 0.6 mm. Surfaces: entire face ME: 0.81 +/- 0.22 mm, for UL: 1.2 +/- 0.6 mm, LL: 1.4 +/- 0.5 mm, chin: 1.1 +/- 0.6 mm; Error equal or < 1 mm: 83.3%, < 2 mm: 100%. ME among patients who had a V-Y closure significantly smaller than those without a V-Y closure.
2015b, Liebregts	RS	100	31.6^^	F: 65, M: 35	C II	CBCT	No	Maxilim v.2.2.2.1 - MTM	BSSO	Landmarks: ME at SN 1.1 +/- 0.5 mm, at LS 1.5 +/- 0.7 mm, at LI 2.0 +/- 1.0 mm, at sublabiale 1.7 +/- 1.1 mm , at Pog 1.5 +/- 0.9 mm. Surface: entire face ME: 0.9 +/- 0.3 mm; error equal or < 2 mm: 100%, <1 mm: 78%. ME for UL 0.9+/-0.5, LL 1.2+/-0.5, and chin 0.8 +/- 0.5mm. Average absolute error less or equal to 2 mm for UL: 98%, for LL 94% , and for the chin 97%
2015, Van Hemelen	PS	31	*	*	C II, C III	CBCT	No	Maxilim - MTM	BSSO, BSSO+Ch, LFI, LFI+Ch, BIMAX, TRIMAX	ME in the horizontal direction: 1.48 mm, in the vertical direction: 1.46 mm
2016, Liebregts	RS	60	26	F: 45, M: 15	NR	CBCT	No	Maxilim - MTM	BIMAX	ME: 1.0 +/-0.9 mm in alar width
2016, Resnick	RS	7	18.1 ± 1.0	F: 5, M: 2	MH	CBCT	Yes[c]	Dolphin 3D v.11.8 - sparse landmark-based algorithm	LFI	ME: 2.91 +/- 2.16 mm, for midline points: 1.66 +/- 1.82 mm, for lateral points: 3.84 +/- 1.92 mm. 2 (33%) midline points with error > 2 mm (SN, SA), 6 (75%) lateral points > 2 mm. ME at NLA: 8.1 +/- 5.6 degrees

2017, Kim	RS	40	22.5 ± 3.2	F: 22, M: 18	C I, C II, C III	MSCT**	Yes[c]	FEM with the mucosa sliding effect	BIMAX, TRIMAX	Quantitative: entire face ME 1.1 +/- 0.3mm, UL 1.2 +/- 0.7 mm, LL 1.5 +/- 0.7 mm, chin 1.3 +/- 0.7. Qualitative: 80% (32/40) clinically acceptable
2021, Kim	RS	35	23.0 ± 4.0	F: 17, M: 18	C I, C II, C III	MSCT**	Yes[c]	FEM with the sliding effect of the lip and the mucosa	BSSO, BIMAX, TRIMAX	Quantitative: entire face ME 1.03 +/- 0.30 mm, UL 0.86 +/- 0.36 mm, LL 1.10 +/- 0.41 mm, chin 1.08 +/- 0.51mm. Qualitative: improvement in lips compared with previous FEM methods
2017, Mundluru	RS	13	NR	NR	As	CBCT	No	Maxilim - MTM	BIMAX, BSSO, BSSO+Ch	Underprediction of ST changes. The signed ME from -0.55 to 0.43 mm; The absolute ME from 0.6 to 1.3 mm
2018, Holzinger	PS	16	26	F: 8, M: 8	OB: C II, C III	MSCT	No	SOTIRIOS	NR - surgery first	ME: 1.46 +/- 1.53 mm; 50% < 1.03 mm, 80% < 2.20 mm, and 95% up to 4.34 mm
2019, Knoops	RS	7	18 ± 1	F: 5, M: 2	MH	CBCT	No	(1)Dolphin 3D v.11.95 - sparse landmark-based algorithm; (2)ProPlan CMF v.3.0.1 - FDM; (3)PFEM	LFI	RMSDolphin = 1.8 +/-0.8 mm, RMSPro-Plan = 1.2+/-0.4 mm, and RMSPFEM = 1.3+/-0.4 mm; average percentage of points <2 mm: PDolphin = 83+/-12%, PProPlan = 91+/-9%, and PPFEM = 88+/-10%. Better results for ProPlan and PFEM compared to Dolphin
2019, Elshebiny	RS	20	22.7 ± 2.1	F: 11, M: 9	NR	CBCT	No	Dolphin 3D v.11.9 - sparse landmark-based algorithm	BIMAX/TRIMAX	Statistically significant differences in 2 angular measurements (FNA, NLA) and in 3 linear measurements (SA, UL length and subalar width)
2021, Cunha	RS	16	36	F: 11, M: 5	C II	MSCT**	No	OrtogOnBlender-OOB - MSM	BIMAX/TRIMAX	ME for all landmarks < 2 mm, entire face ME: 1.07mm . MaxE: ChR, ChL, and SB
2021, Willinger	NR	19	22	F: 5, M: 14	midfacial deficiency, C III	MSCT/ CBCT**	No	(1)IPS Case Designer - MTM; (2)Dolphin 3D v.11.95 - sparse landmark-based algorithm	modified IQLFIIO +/- BSSO	IR level: Dolphin ME 2.90 +/- 2.1mm, IPS ME 1.70 +/- 1.3mm; SF level: Dolphin ME: 3.57 +/- 2.0mm, IPS ME: 1.34 +/- 0.9mm; LI level: Dolphin ME 2.48 +/- 1.9mm, IPS ME 2.25 +/- 1.6mm. MaxE for Dolphin at SF level
2021, Tanikawa	RS	72	23.5	NR	C III	No – lat ceph	Yes	Geometric morphometric methods (GMM), DL	BIMAX	The system error 0.89 ± 0.30 mm. MaxE of 0.8–1.2 mm in the nasal ala, chin, corner of the mouth; the total success rate at < 1 mm: 54%; and at < 2 mm: 100%
2021, ter Horst	RS	14	34.2 ± 13.0	F: 11, M: 3	C II	CBCT	Yes[c]	DL; IPS CaseDesigner - MTM	BSSO	<u>DL-based</u>: The lower face ME: 1.0 +/- 0.6 mm, simulations with MaxE of 1 mm: 64.3% and of 2 mm: 92.9%. RMS 1.2 +/- 0.6 mm; ME for LL 1.1 +/- 0.9 mm; for the chin 1.4 +/- 0.9 mm. <u>MTM-based</u>: The lower face ME: 1.5 +/- 0.5 mm, simulations with MaxE of 1 mm: 21.4% and of 2 mm: 85.7%. RMS 2.0 +/- 0.7 mm; ME for LL 1.7 +/- 0.9 mm; for the chin 2.0 +/- 1.0 mm. The DL model with higher accuracy
2021, Alcañiz	RS	10	32	F: 8, M: 2	C II, C III, As, OB	CBCT**	Yes**	FEM	LFI, LFII, BSSO, USSO, Ch, BIMAX	Surface with error < 3mm with coarse meshes: 92%, with fine meshes: 95%
2022, Lee	RS	10	NR	NR	C III	CBCT**	Yes**[c]	ProPlan CMF - FDM	BIMAX	Entire face ME: 0.73 +/- 0.21mm, for LL: 1.42 +/- 0.77mm, for UL: 1.14 +/-0.80mm, for chin: 0.95 +/-0.58mm; error < 2mm: 90.9%
2022, Gutiérrez	RS	10	32	F: 8, M: 2	C II, C III, As, OB	CBCT**	Yes**	FEM	LFI, LFII, BSSO, USSO, Ch, BIMAX	All distances for both meshes and their mean distances significantly < 2 mm, except LL, RGo and LGo. Distances for all landmarks significantly < 3 mm, except for LL of the fine mesh
2022, Yamashita	RS	88	NR^	F: 62, M: 26	C II, C III	CBCT	No	Dolphin 3D v.11.95 - sparse landmark-based algorithm	BIMAX, TRIMAX	C II: underprediction with downward direction in S-Y, S-Z, LI-Y, SB-Y, Pog-Y, Pog-Z, Gn-Y, Gn-Z, Me-Y, Me-Z, values > 2 mm: LI-Y, SB-Y, Pog-Y, Gn-Y, Gn-Z, Me-Y. MaxE LI-Y: 2.73 mm. C III: overprediction and downward direction in Pog-Z, Gn-Y, Gn-Z, Me-Y, and Me-Z, all discrepancies < 2 mm.

2022, Ma	NR	40	NR^	F: 24, M: 16	NR	MSCT**	No	FSC-Net, point cloud DL	NR	Qualitative: FSC-Net comparable with FEM-RLSE. Quantitative: landmarks entire face ME 2.95 +/-0.61mm; surface entire face ME: 1.55 +/-0.30mm, lips 1.58 +/- 0.26mm, chin: 2.11 +/-0.77mm; FSC-Net comparable with FEM-RLSE
2022, Awad	NR	20	27.3	F:10, M:10	C II, C III	CBCT	Yes	IPS CaseDesigner v.2.1.4.4 - MTM	BIMAX	Entire face ME: -1.5 to 1.4 mm, UL -2.5 to 1.3 mm, LL -2.1 to 2.5 mm, chin -1.8 to 2.6 mm
2022, Hou	RS	58	26.7	F: 37, M: 21	C III	CBCT	Yes[c]	ProPlan CMF - FDM	BIMAX	Entire face ME: 1.43 +/- 0.40 mm. Error of UL, LL, chin, right external buccal and left external buccal > 2.0 mm; LL the least predictable: 2.69 ± 1.25 mm
2023, Şenyü-rek	RS	16	18.5 ± 2.13	NR	MH	CBCT**	No	ProPlan CMF v.3.0 - FDM	LFI	Error in UL and LL: 1.49 +/- 0.77 mm, in cheeks: 0.98 +/- 0.34 mm, nose: 0.86 +/- 0.23 mm, and eyes: 0.76 +/- 0.32 mm
2023, Ruggiero	PS	5	NR	NR	NR	CBCT + MRI	No	FEM with patient-specific model generated from CBCT and MRI	BIMAX	Midface ME: 0.55 +/- 2.29 mm
2024, Fang	RS	40	NR	NR	NR	MSCT**	No	DL, ACMT-Net with the CPSA module	BIMAX	Quantitative: Surface entire face ME 1.06 +/- 0.43 mm, UL 1.13 +/- 0.71 mm, LL 1.23 +/- 0.48 mm, chin 1.13 +/- 0.62mm; landmarks entire face ME 2.44 +/- 0.45 mm, upper face 1.23 +/- 0.47 mm, lower face 3.25 +/- 0.66 mm Qualitative: 77.5% (31/40) of the simulations clinically acceptable

^ only range was reported; ^^ growing patients also included in the study group < 14yo; *not clear; ** device not specified; [a] details in the appendix D; [b] most relevant results; [c] 3D photograph fused with the MSCT/CBCT skin surface; As, asymmetry; BIMAX, bimaxillary osteotomy; BSSO, bilateral mandibular sagittal split osteotomy; Ch, genioplasty; ChL, cheilion left; ChR, cheilion right; C, Class; ComL, left commissure; ComR, right commissure; DL, deep learning; F, female; FDM, finite difference method; FEM, finite element model; FNA, frontonasal angle; Gn, soft tissue gnathion; IR, infraorbital rim; IQLFIIO, intraoral quadrangular Le Fort II osteotomy; lat ceph, lateral cephalograms; LFI, Le Fort I maxillary osteotomy; LGo, soft tissue left gonion; Li, crown of the lateral incisor; LI, labrale inferior; LL, lower Lip; LS, labrale superior; M, male; MaxE, maximal error; Me, soft tissue menton; ME, mean error; MH, maxillary hypoplasia; MinE, minimal error; MSM, mass spring model; MTM, mass tensor model; NLA, nasolabial angle; NR, not reported; OB, open bite; PFEM, probabilistic FEM; Pog, soft tissue pogonion; PS, prospective study; RGo, soft tissue right gonion; RMS, root mean square distance; RS, retrospective study; S, stomion; SA, soft tissue A point; SB, soft tissue B point; SF, sinus floor; SN, subnasale; ST, soft tissue; TRIMAX, bimaxillary osteotomy and genioplasty; UL, upper Lip; USSO, unilateral mandibular sagittal split osteotomy. C I, correct relationship between the maxilla and the mandible. C II, retrusive mandible in relation to the maxilla; C III, protrusive mandible in relation to the maxilla.

Table S3. The revised Quadas2 tool for risk of bias and applicability assessment.

**Study**	**Risk of Bias**				**Applicability**		
	**Patient selection**	**Index Test**	**Reference Standard**	**Flow and Timing**	Patient selection	Index Test	Reference Standard
2004, Chabanas	☹	😎	😎	☹	😐	☹	😎
2007, Mollemans	😐	😎	😎	☹	😎	☹	😎
2007, Marchetti	😎	☹	😎	☹	☹	☹	😎
2010, Bianchi	😐	😎	☹	☹	😎	☹	😎
2010, Ulusoy	😐	☹	😎	😐	😐	☹	😎
2011, Centenero	😐	☹	😎	☹	😎	☹	😎
2011, Marchetti	😎	😎	😎	☹	☹	☹	😎
2013, Schendel	☹	☹	😎	☹	☹	☹	😎

2013, Shafi	Low	Low	Low	Low	High	Low	Low
2013, Nadjmi	Low	High	Low	High	Low	High	Low
2014, Terzic	Low	High	Low	High	Unclear	High	Low
2014, Nadjmi	Low	Low	Low	High	High	High	Low
2015, Ullah	Low	Low	Low	Low	High	Low	Low
2015, Khambay	Unclear	Low	Low	Unclear	High	Low	Low
2015, Nam	Low	High	Low	Unclear	High	Low	Low
2015a, Liebregts	Low	Low	High	Unclear	High	Low	Low
2015b, Liebregts	Low	Low	High	Low	High	Low	Low
2015, Van Hemelen	Unclear	High	Low	High	Low	High	High
2016, Liebregts	Low	Low	High	Low	High	High	Low
2016, Resnick	Low	Low	High	High	High	Low	Low
2017, Kim	Low	Low	Low	High	Low	Unclear	Low
2021, Kim	Low	Low	Low	High	Low	Unclear	Low
2017, Mundluru	Unclear	Low	Low	Unclear	High	Low	Low
2018, Holzinger	Low	Low	Low	High	Unclear	High	Low
2019, Knoops	Low	Low	High	Low	High	Low	High
2019, Elshebiny	Low	Low	Low	High	Unclear	High	Low
2021, Cunha	Low	High	Low	High	High	Low	Low
2021, Willinger	Low	Low	Low	High	High	High	Low
2021, Tanikawa	Low	Low	High	Low	High	High	Low
2021, ter Horst	Low	Low	Low	High	High	Low	Low
2021, Alcañiz	High	Low	Low	Unclear	Low	High	Low
2022, Lee	Unclear	Low	High	High	High	Low	Low
2022, Gutiérrez	High	Low	Low	Unclear	Low	High	Low
2022, Yamashita	Low	High	Low	Low	High	High	Low
2022, Ma	Low	High	Low	Unclear	Unclear	Low	Low
2022, Awad	Low	High	Low	High	Low	Low	Low
2022, Hou	Low	Low	High	High	Low	Low	High
2023, Şenyürek	Unclear	Low	Low	Unclear	High	High	Low
2023, Ruggiero	Unclear	Low	Low	High	Unclear	High	Low
2024, Fang	Unclear	Low	Unclear	High	Unclear	Unclear	Low

Low risk Unclear risk High risk.

Table S4. Image acquisition technique and device.

Year, author	Image acquisition	
	MSCT/CBCT device	3D photograph device
2004, Chabanas	MSCT *	
2007, Mollemans	MSCT *	3D camera system (Eyetronics, Leuven, Belgium)
2007, Marchetti	MSCT - High Speed Spiral (GE,Waukesha, WI)	

2010, Bianchi	CBCT - NewTom 3G (QR, Verona, Italy)	
2010, Ulusoy	MSCT*	
2011, Centenero	MSCT - General Electric HiSpeed; CBCT - IS I-CAT 17-23	
2011, Marchetti	MSCT - 16 slices Light Speed (GE, Milwaukee, WI, USA)	
2013, Schendel	CBCT - i-CAT (Imaging Sci. Int., Hatfield, PA, USA)	3dMD (Atlanta, GA, USA)
2013, Shafi	CBCT - i-CAT (Imaging Sci. Int., Hatfield, PA, USA)	
2013, Nadjmi	CBCT*; CBCT synthesized lateral cephalograms	*
2014, Terzic	MSCT - Siemens Sensation 64; (Germany) / CBCT - NewTom VGi (QR, Verona, Italy)	3dMDTrio System (3dMD, Atlanta, GA, USA)
2014, Nadjmi	CBCT - i-CAT (Imaging Sci. Int., Hatfield, PA, USA)	
2015, Ullah	CBCT - i-CAT (Imaging Sci. Int., Hatfield, PA, USA)	
2015, Khambay	CBCT*	
2015, Nam	MSCT - GE lightspeed VCT XT(GE,Milwaukee, WI, USA)	
2015a, Liebregts	CBCT - i-CAT (Imaging Sci. Int., Hatfield, PA, USA)	
2015b, Liebregts	CBCT - i-CAT (Imaging Sci. Int., Hatfield, PA, USA)	
2015, Van Hemelen	CBCT - i-CAT (Imaging Sci. Int., Hatfield, PA, USA)	
2016, Liebregts	CBCT - i-CAT (Imaging Sci. Int., Hatfield, PA, USA)	
2016, Resnick	CBCT: i-CAT (Imaging Sci. Int., Hatfield, PA, USA) /Planmeca Promax 3D Max (Planmeca, Roselle, IL, USA)	3D VECTRA M3 (Canfield Scientific, Fairfield, NJ, USA)
2017, Kim	MSCT*	3dMD (Atlanta, GA, USA)
2021, Kim	MSCT*	3dMD (Atlanta, GA, USA)
2017, Mundluru	CBCT - iCAT (Imaging Sci. Int., Hatfield, PA, USA)	
2018, Holzinger	MSCT - Philips Brilliance 64 (Amsterdam, NL)	
2019, Knoops	CBCT: i-CAT (Imaging Sci. Int., Hatfield, PA, USA) /Planmeca Promax 3D Max (Planmeca, Roselle, IL, USA)	
2019, Elshebiny	CBCT CB Mercuray (Hitachi Medical Systems America, Twinsburg, OH)	
2021, Cunha	MSCT*	
2021, Willinger	MSCT/CBCT*	
2021, Tanikawa	lateral cephalograms	3dMD cranial System (3dMD, Atlanta, GA, USA)
2021, ter Horst	CBCT - i-CAT (Imaging Sci. Int., Hatfield, PA, USA)	3dMD face System (3dMD, Atlanta, GA, USA)
2021, Alcañiz	CBCT*	*
2022, Lee	CBCT*	*
2022, Gutiérrez	CBCT*	*
2022, Yamashita	CBCT - i-CAT (Imaging Sci. Int., Hatfield, PA, USA)	
2022, Ma	MSCT*	
2022, Awad	CBCT - KaVo 3D (KaVo Dental GmbH; Biberach an der Riß, Germany)	3dMDTrio System (3dMD; Brentford, London, UK)
2022, Hou	CBCT - NewTom (NewTom AG, Marburg, Germany)	3dMDTrio System (3dMD, Atlanta, GA, USA)
2023, Şenyürek	CBCT *	
2023, Ruggiero	CBCT - NewTom 3000 VGI Evo (Cefla Group, Imola, Italy), MRI – GE 1.5 T	
2024, Fang	MSCT*	

* device not specified.
